# Stress Induced by Fishing in Common Octopus (*Octopus vulgaris*) and Relative Impact on Its Use as an Experimental Model

**DOI:** 10.3390/ani15040503

**Published:** 2025-02-11

**Authors:** Valeria Maselli, Mariangela Norcia, Bruno Pinto, Emanuela Cirillo, Gianluca Polese, Anna Di Cosmo

**Affiliations:** 1 Department of Biology, University of Naples Federico II, Via Cinthia, 80126 Naples, Italy; valeria.maselli@unina.it (V.M.); mariangela.norcia@unina.it (M.N.); bruno.pinto@szn.it (B.P.); emanuela.cirillo@unina.it (E.C.); gianluca.polese@unina.it (G.P.); 2 Department of Ecosustainable Marine Biotechnology, Stazione Zoologica Anton Dohrn, Via Ammiraglio Ferdinando Acton 55, 80133 Naples, Italy; 3 Department of Ecosustainable Marine Biotechnology, Stazione Zoologica Anton Dohrn, Ischia Marine Centre, 80077 Ischia, Italy; 4 MNESYS—PNRR Partenariato Esteso, 16132 Genova, Italy

**Keywords:** *Octopus vulgaris*, fishing stress, animal welfare, artisanal fishing, research ethics, welfare biomarkers

## Abstract

The common octopus (*Octopus vulgaris*), among coleoid cephalopods, has evolved the most complex nervous system and sophisticated behaviors. Historically, *O. vulgaris* was a key animal model for neurophysiology research, and today, it is studied for its genomic innovations. However, unlike other models, there is no octopus farming for research, so specimens must be collected from the wild. This study assessed the impact of fishing on octopuses used in research, considering those caught using artisanal pots in the ‘Regno di Nettuno’ Marine Protected Area, Ischia (NA). To evaluate fishing stress, we identified morphological stress indicators such as chromatophore pattern and posture, and three potential molecular markers, estrogen receptor (ER), catalase (CAT), and heat shock protein (HSP70). We measured the percentage of stress signals shown by fished specimens and analyzed their differential gene expression. The transcriptional levels of octopuses caught using traps were compared to control specimens acclimated in captivity. Results indicated fluctuations in gene expression due to fishing stress. These findings suggest that an acclimation period after the stress event of fishing is crucial for ensuring the welfare of octopuses used in research, thus enhancing the quality of physiological and ethological studies.

## Simple Summary

The common octopus (*Octopus vulgaris*) is a fascinating marine animal known for its advanced nervous system and complex behaviors, making it an important model for both ethological and molecular research. Unlike other research animals like zebrafish, octopuses are not farmed, so they must be collected from the wild for studies. This study investigated how fishing impacts the welfare of *O. vulgaris* used in research. We compared *O. vulgaris* caught using artisanal pots in the ‘Regno di Nettuno’ Marine Protected Area with individuals subsequently kept under controlled conditions. We examined both morphological stress signals and the expression of stress-related genes—estrogen receptor, catalase, and heat shock protein—and we found that fishing induces significant changes in gene expression, reflecting physiological stress. Our main goal was first to evaluate the stress impact of fishing on the octopuses using a new set of welfare biomarkers. The second is to improve the octopuses’ welfare, supporting the researchers in their behavioral studies and avoiding data misinterpretation due to the stress impact of fishing. These findings emphasize the need for an appropriate evaluation of the animals using welfare biomarkers after fishing to improve their welfare and ensure more reliable scientific results.

## 1. Introduction

Cephalopods are marine animals inhabiting all oceans, able to colonize various habitats, from coastal to deep sea, and compete with vertebrates successfully [1].

Cephalopods’ complex behavioral and learning capabilities are supported by a highly sophisticated nervous system [2,3]. Among the cephalopods, the coleoid *Octopus vulgaris*, the common octopus, has a huge nervous system (500 million neurons) with almost 40 different lobes controlling a wide range of functions and axial nerve cords in its arms [4,5]. The central ‘brain’ is formed of more than 24 lobes surrounding the esophagus and controlling some sensory perceptions and behaviors. About 300 million neurons diffusely distributed along the arms innervate the muscles and an enormous number of suckers to coordinate movements and acquire tactile, visual, and chemosensory signals [5,6].

Cephalopods, particularly octopuses used in research, come from the wild and are provided by fishermen. For all octopus species, aquaculture potential has been investigated in recent years, including *Octopus maya* [7], *Octopus bimaculoides* [8], or *O. vulgaris* [9,10,11,12,13,14]; commercial-scale farming of *O. vulgaris* has recently begun in Spain and some Asian countries. However, these farms primarily target the food industry, and their accessibility for research purposes remains limited. Meanwhile, other animal models, such as mice or zebrafish, are bred for research in controlled animal houses.

In Italy, the fishing of *O. vulgaris* has been regulated since 2018, and current European regulations (EU Regulation 2019/1241) do not specify a minimum landing size, marketing size, or fishing techniques for *O. vulgaris*. Additionally, a substantial portion of caught octopus comes from recreational fishing, such as spearfishing, which remains largely unregulated. Currently, fishing for *O. vulgaris* occurs year-round using a variety of techniques, including trawling, traps, pots, and gill nets [10,14,15,16,17,18,19,20,21,22].

The fishing methods used to capture *O. vulgaris* often fail to fully comply with European cephalopod protection regulations, raising concerns about the sustainability of these practices. Traditional gear, such as artisanal traps and non-compliant gillnets, can cause significant injuries such as mantle abrasions and arm mutilations resulting in stress for the animals. Acute stress in *O. vulgaris* has been previously described, considering survival rates and physiological, functional, and molecular immune responses [21,23,24].

This study was conducted in the ‘Regno di Nettuno’ Marine Protected Area (MPA-RdN), which includes the islands of the Flegreo Archipelago (Ischia, Procida, and Vivara) in the Gulf of Napoli (FEAMP 2014–2020).

Our main goal was first to evaluate the stress impact of artisanal fishing on the octopuses that will be used in research using a new set of welfare biomarkers. The second is to improve the octopuses’ welfare, supporting the researchers in their behavioral studies and avoiding data misinterpretation due to the stress impact coming from fishing.

This novel approach will combine data coming from the evaluation of morphological stress indicators exhibited by the animals immediately after fishing with data from three gene expression levels: estrogen receptor (ER), catalase (CAT), and heat shock protein (HSP70) are involved in hormonal, oxidative, and thermal stress, respectively [25,26,27,28,29,30,31,32,33,34,35,36,37,38].

The present study identified potential morphological and molecular welfare biomarkers that could be utilized in studies of stressful conditions in the common octopus.

## 2. Materials and Methods

### 2.1. Sampling and Morphological Observations

A small-scale fishery operating in MPA-RdN was selected for a periodic survey. The survey aimed to assess the number of octopuses caught, the fishing effort, the type and number of traps utilized, and the exact location and depth at which they were dropped down. Octopuses were collected using cylindrical creels, supported by a metal or plastic frame with rigid 1 cm mesh, lowered into the sea at depths ranging from 2 to 70 m, often in series of 80 to 100 traps spaced about 8 m apart. The traps were baited with sardines, crabs, or discarded fish, and checked every 12 to 48 h, depending on weather conditions.

A total of 29 specimens of *O. vulgaris* (149–1660 g) were sampled during the winter period (December 2021 and January 2022), which was chosen to avoid the breeding season of *O. vulgaris*. This choice was made to minimize physiological variations associated with reproductive activity, which could affect the markers analyzed. These specimens were randomly selected among the daily commercial landings. For each of them, morphometric data about sizes (Table 1) and morphological stress indicators such as vitality, presence/absence of mantle bruises, and arm mutilations were collected. For vitality assessment, the following behavioral parameters were observed: visual response to the operator’s presence, response to tactile stimulation, response to feeding, and changes in color patterns [39]. The sex was assessed by observing the third right arm and hence the presence (or the absence) of the ectocotile, which indicates the male phenotype. In cases of third right arm absence, possibly due to injuries or mutilation after a fight, the sex was evaluated by dissection.

A subset of 16 octopuses was chosen, ensuring a balanced distribution among samples with comparable size, vitality, and mutilations/mantle bruises. They were transferred to Di Cosmo’s cephalopod facility at the Department of Biology, University of Naples Federico II (Italy) Octopus CORE (https://shorturl.at/bvZbb (accessed on 30 June 2024)), in dark thermic tanks filled with seawater and connected to a mobile aeration system. We considered 12 animals as the fished group (6 mature males and 6 mature females) [25,40,41], and 4 animals (2 mature males and 2 mature females) as the control group. The specimens belonging to the fished group were immediately anesthetized by isoflurane insufflation preceded by a MgCl_2_ bath [42]. The arm tips and gonads were dissected in sterile conditions. All used males presented spermatophores in their reproductive tract, while females were between the previtellogenic and early vitellogenic phases [25].

Control animals were housed in the cephalopod facility, they were kept individually in large fiberglass tanks (50 × 50 × 50 cm) filled with circulating seawater [39]. Water temperature was kept at 18 ± 1 °C (mean ± SD), and illumination was maintained with natural photoperiod. All tanks were enriched by adding an amphora (as a den) and rocks (about 6 cm^3^). The control animals were acclimatized for 15 days [43]. During this time, octopuses were fed ad libitum (anchovy, *Engraulis encrasicolus*; clam, *Ruditapes philippinarum*; mussel, *Mytilus edulis),* and several physiological and behavioral parameters were monitored to verify the welfare and healthiness of the octopuses [39,43].

To ensure that the animals exhibited no signs of stress and were fully acclimatized, we assessed their chromatophore pattern, posture, predatory strategy, and problem-solving abilities [2]. The problem-solving ability of *O. vulgaris* was evaluated through experiments conducted for 3 consecutive days per week, during which the animals were presented with two plastic jars closed with a screw lid containing live prey. During this period, the octopuses could only feed by opening the jars [43].

After acclimatization, octopuses were anesthetized by isoflurane insufflation preceded by a MgCl_2_ bath [42]. The arm tips and gonads were dissected in sterile conditions for molecular analysis.

For taxonomic molecular species identification, we performed DNA analysis, and the arm tips were placed in 70% ethanol and stored at −20 °C.

For RNA gene expression analysis, gonads were snap-frozen, placed in TRIzol (Invitrogen, Thermo Fisher Scientific Inc., Waltham, MA, USA), and stored at −80 °C.

All procedures were conducted following European Directive 2010/63/EU and Italian Legislative Decree 26/2014. The study adhered to the ethical principles of the 3Rs— reduction, refinement, and replacement—(Project n 608/2016-PR-17/06/2016; protocol n°DGSAF 0022292-P-03/10/2017).

### 2.2. Taxonomic Molecular Species Identification

The genomic DNA was extracted from the arm tips using the DNeasy Blood & Tissue Kit (QIAGEN, Hilden, Germany) according to the manufacturer’s instructions. The quality and amount of purified DNA were analyzed spectrophotometrically with Nanodrop2000 (Thermo Scientific Inc., Waltham, MA, USA). A fragment of the mtDNA cytochrome c oxidase subunit I (COXI) region was amplified using the primers LCO1490 (5′-GGT CAA CAA ATC ATA AAG ATA TTG G-3′) and HCO2198 (5′-TAA ACT TCA GGG TGA CCA AAA AAT CA-3′) [44].

Polymerase chain reactions (PCRs) were set up in 25 µL volumes containing the following: 25 ng of DNA template, 1 U of Taq DNA polymerase (QIAGEN, Germantown, MD, USA), 2.5 µL of 10X PCR buffer, 2.5 mM MgCl_2_, 0.25 mM of each deoxynucleotide triphosphate (dNTP), and 12.5 pmol of each primer. The amplification was performed in a Mastercycler (Eppendorf, Hamburg, Germany) under the following conditions: an initial DNA denaturation at 95 °C for 3 min and 40 cycles at 94 °C for 30 s, 62 °C for 40 s, 72 °C for 1 min, and a final elongation step at 72 °C for 10 min. Amplifications of blank extractions and PCR blanks were performed in all experiments to monitor the contamination. PCR products in quantities of 5 µL were then separated by electrophoresis in a 1.5% agarose gel. PCR products were sequenced in both directions (Eurofins Genomics GmbH, Ebersberg bei München, Germany). Electropherograms were assembled to examine any base pair ambiguities, and the new sequences were aligned using the software Geneious version Prime 2024.0 (Biomatters, http://www.geneious.com/ (accessed on 14 February 2024)). Sequences were analyzed with GenBank BLASTn and BLASTx (BLAST, basic local alignment search tool).

### 2.3. RNA Gene Expression Analysis for Estrogen Receptor (ER), Catalase (CAT), and Heat Shock Protein (HSP70)

Total RNA was extracted using the Direct-zol RNA Miniprep Plus Kit (Zymo Research, Irvine, CA, USA) according to the manufacturer’s instructions. The quality and amount of purified RNA were analyzed spectrophotometrically with Nanodrop2000 (Thermo Scientific Inc., Waltham, MA, USA). Subsequently, 1000 ng of RNA was reverse-transcribed into cDNA using the QuantiTect^®^ Reverse Transcription Kit (QIAGEN, Hilden, Germany). Specific PCR primers were designed using Geneious version Prime 2024.0 (Biomatters, http://www.geneious.com/ (accessed on 14 February 2024)), targeting the coding sequence for estrogen receptor (ER) designed on the *O. vulgaris* genome, catalase (CAT) [26], and heat shock protein (HSP70) [26], with Ubiquitin Ribosomal Protein S27a used as a control [6] (Table 2). 

### 2.4. Statistical Analysis

Quantitative RT-PCR analysis was conducted by using the 2^−(ΔΔCt)^ method [45]. At the end of each test, a melting curve analysis was carried out (plate read every 0.5 °C from 55 to 95 °C) to determine the formation of the specific products. RT-PCRs were run in triplicate and blanks were performed in all experiments to monitor the contamination. Results coming from the same group are pulled together. Males and females of control and the fished groups were compared and analyzed using Student’s *t*-test. Data with *p*-values < 0.05 were considered statistically significant.

## 3. Results

### 3.1. Morphometric Data Analysis

Morphometric measures and their sex identification were taken for all 29 collected animals and are reported in Table 3. 16 individuals were selected for gene expression studies (Table 3, dark gray = fished group; light gray = control group).

**Figure 1:**
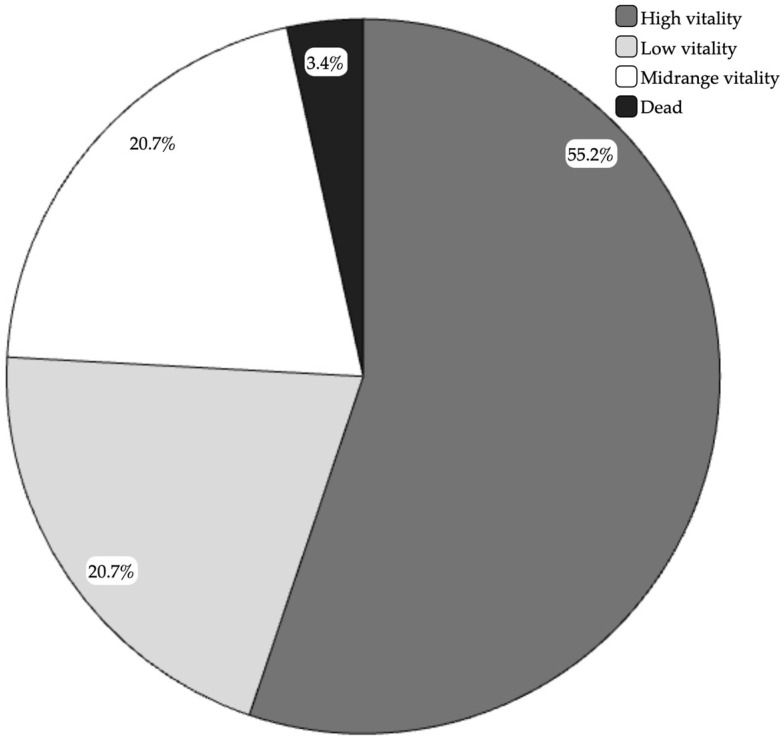
Health status of fished octopuses. The graph shows the different percentages of fished octopuses based on their health status: in light blue, 55.2% of animals with high vitality rate and no or light mantle/arm damage (16 animals used for the study); in yellow, 20.7% of animals with low vitality rate and presence of mantle bruises and/or one arm injured; in green, 20.7% of animals with midrange vitality rate, mantle bruises and more than one arm injured; in purple, 3.4% of the animals fished dead.

The observations of morphological stress indicators revealed that 55.2% of the animals (16 animals used for the study) had a high vitality rate with light or no mantle/arm damage; 20.7% of animals had a low vitality rate with the presence of mantle bruises and one arm injured; 20.7% had a midrange vitality rate with mantle bruises and more than one arm injured; just 1 animal, corresponding to 3.4% of the total, was fished dead (Figure 1).

### 3.2. Taxonomic Molecular Species Identification and RNA Gene Expression Analysis for Estrogen Receptor (ER), Catalase (CAT), and Heat Shock Protein (HSP70)

**Figure 2:**
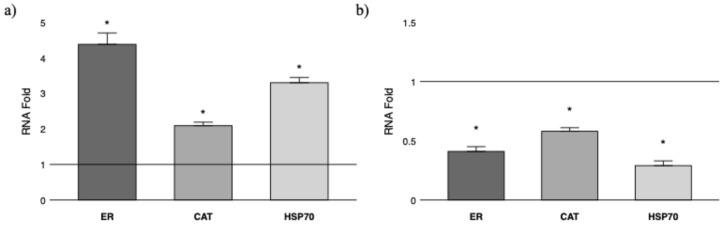
Gene expression analysis in gonads for ER, CAT, and HSP70 in fished *O. vulgaris* male (**a**) and female (**b**). Relative mRNA fold change in gene expression is compared to the relative control group (control males N = 2, control females N = 2, fished males N = 6, fished females N = 6; control line set y = 1; control males SEM = 0.01; control females SEM = 0.01). * asterisk indicates that the difference vs. control group is statistically significant (*p* < 0.05). Error bars represent the SEM.

The sequencing analysis confirmed that all collected samples belonged to the *O. vulgaris* species and the identity of the ER, CAT, and HSP70 genes. Subsequently, we analyzed male and female fished samples separately, focusing on relative mRNA fold change normalized against the gene expression of control samples (Figure 2).

In males (Figure 2a), the ER gene showed a significantly elevated expression, approximately 4.38 times higher than the control. The CAT gene expression increased by about 2.09 times, while HSP70 was upregulated around 3.3 times compared to the control, with statistical significance. In females (Figure 2b), the expression levels of all three genes were downregulated compared to the control. The ER gene had the lowest expression, around 0.41, followed by CAT at 0.58, while HSP70 showed a similar expression level to CAT, which was statistically significant.

### 3.3. Behavioral Acclimatization Data

All the animals in the control group, by the end of the acclimation period, which lasted 15 days, exhibited a normal chromatophoric pattern, physiological posture, appropriate predatory strategy, and effective problem-solving behavior. All acclimatized individuals of the control group opened the two screw-lid jars within the third day, showing learning and adaptation.

## 4. Discussion

Cephalopods, particularly *O. vulgaris*, represent a research animal model for ethological and molecular studies due to their unique neurobiological characteristics and ability to adapt to different environmental conditions [46]. Their welfare is an important and timely issue to ensure their health and the availability of specimens for research [47]. To achieve optimal results, it is essential to work with animals that do not reflect the signs of fishing stress [22,27]. Stressful conditions can alter the physiology and behavior of the specimens, affecting experimental results [15,19,40,42,43,46].

In addition, ensuring responsible fishing practices and collaborating with local fishermen who adopt sustainable practices is critical to minimize the effect of fishing stress on experimental data. In fact, more than 50% of fished octopuses bear bruises on their mantle, and 41.4% have one or two mutilated arms. When octopus specimens show such morphological stress signals, they should not be used for research purposes because they could give altered physiological and ethological results. Several welfare markers useful in monitoring and minimizing animal stress have been proposed to address the stress problem and ensure the welfare of octopuses used in research [21].

In this study, we identify three other molecular markers that can determine the stress due to fishing and transfer practices: estrogen receptor (ER), catalase (CAT), and heat shock protein (HSP70).

Octopus ER is widely expressed in males and females in different tissues, including the nervous system, the testes, and the ovaries [25,48,49,50,51,52]. In the octopus female, the reproductive system is the source of sex hormones, showing morphological changes for egg maturation and production [25,49,53]. Previously it has been demonstrated that estrogen concentrations fluctuated throughout life stages [25]. Stress affects estrogen receptor levels, which could disrupt critical reproductive events, causing a suppression in reproduction [27]. A previous study on stress, simulating fishing activities by chasing *O. bimaculoides* for 5 min, showed that sex hormones are affected by stress events only in males during the reproductive stage [28].

Catalase is an antioxidant enzyme that catalyzes the decomposition of hydrogen peroxide (H_2_O_2_) into water and molecular oxygen [29]. This enzyme is present in almost all aerobic organisms, including many invertebrates, and plays a crucial role in the defense against oxidative stress, protecting cells from oxidative damage that can impair their function [30]. In invertebrates, catalase is particularly important in maintaining cellular redox balance, especially under adverse environmental conditions, such as exposure to changes in temperature and oxygen, which can increase levels of reactive oxygen species. The enzymatic activity of CAT is well studied in *O. vulgaris* to evaluate oxidative stress due to pollutants and metals in human-altered coastal areas [31,54]. In this study, for the first time, the CAT gene expression levels were measured to evaluate the fishing and transport stress.

Heat shock protein (HSP) families play a fundamental role in regulating normal protein synthesis within the cell [32]. The HSP70 gene modulates its expression due to various sources of stress that disrupt protein folding, such as heat treatment, exposure to toxic materials, ultraviolet irradiation, and pathogen attack [33,34]. The role of HSPs in the stress defense system has been well documented in mollusks, such as stimulated expression of HSP70 in *Mytilus galloprovincialis* or *Ostrea edulis* by heat shock, bacterial infection, and metal exposures [35,36,37,55]. The HSP70 gene expression was also evaluated in *O. vulgaris* paralarvae treated with cadmium and manganese [26]. Water temperature is an important factor in the growth of *O. vulgaris* [56]. In *O. vulgaris* OvHSP70, OvHSF (heat shock factor), and OvHSBP (heat shock binding protein) genes are associated with stress tolerance mechanisms under high temperatures [38].

Here, we observed a clear dimorphism in gene expression patterns between mature male and female fished octopuses. Mature males showed an upregulation of the three selected genes. Meanwhile, females exhibited a downregulation of them, suggesting a different stress response sex-related.

The upregulation of the ER gene in mature fished males is a clear sign of the upheaval of the steroidogenic pathway, which underlies an anomalous hormonal condition affecting physiological and behavioral reproductive functions. As well as for the ER gene, the CAT gene was upregulated in fished males too, implying a stress reaction to potential oxidative damages that octopuses can face during fishing events. Also, the HSP70 gene resulted upregulated in males, which could be due to the effect of thermal stress occurring during the fishing and transportation procedures. These results are in agreement with Hong and co-authors [38], who found an upregulation of HSP70 after experimental thermal stress (higher temperature).

Conversely, the gene expression levels of the three marker genes chosen resulted in a downregulation in our fished mature female specimens. ERs are generally at their highest levels in mature octopus females [51], so the stress events induced by the fishing and transportation procedures had the opposite trend compared to octopus males, affecting their reproductive activities, downregulating their genes. In line with our findings, Larson and Anderson [57] observed differences in hormonal responses between male and female *Enteroctopus dofleini* based on fecal steroid hormone analysis, suggesting the existence of biological mechanisms underlying sex-specific stress response. The downregulation of CAT and HSP70 genes found in mature females indicates that they are less sensitive to stress events, probably due to their physiological status, which is all focused on reproduction. This funding is in agreement with Hong and co-authors [38], given that their specimens were all young and immature.

Currently, there is no size restriction for octopus fishing. Still, considering that the fishing impacts both mature males and females, the capture of big mature females should be avoided a priori to let them go through the reproduction phase.

Furthermore, in scientific research, when possible, the use of male specimens should be preferred, as their collection has a lesser impact on population contraction, which is beneficial for both fishing activities and conservation efforts (FAO, 2020). Fishermen should also be aware of the link between fishing-induced stress and physiological changes in marine organisms, as this stress affects the quality of the catch [58]. Monitoring stress in octopuses is essential to ensure both their health and welfare.

In a recent study, the physiological status and well-being of *O. vulgaris* in captivity were monitored by gene expression analysis in the hemolymph [21]. The hemolymph can be used to analyze the animal’s health status through the expression of genes associated with stress and immune responses. However, variability among individuals and the invasive impact of hemolymph collection on specimens presents a challenge to obtaining reliable and comparable results. Looking ahead, noninvasive techniques such as fecal testing [57] or skin swabs [28] could offer even simpler and more immediate analyses of the physiological status of animals, making monitoring more sustainable [59]. It is, therefore, desirable that future research focus on optimizing these techniques and using the welfare biomarkers proposed here to support the ethical management of *O. vulgaris* in captivity. Moreover, we demonstrate that an acclimation period of 15 days after the stress event of fishing and transportation is essential to minimize the stress effects on behavioral and physiological data and that octopus males are the most suitable candidates as research models given their clearer fluctuation of stress molecular markers, respect to females.

## 5. Conclusions

We identified morphological stress signals and three new key welfare molecular biomarkers (ER, CAT, and HSP70) that are sensitive indicators of stress induced by fishing and transport procedures. An acclimation period of 15 days after the stress event of fishing and transportation, in which individuals opened two screw-lid jars within three days, is essential to ensure reliable results coming from any physiological and ethological experiments. Stress markers revealed that *O. vulgaris* males are more suitable as research models due to their clearer fluctuations in stress-related molecular markers compared to females. This study improves the octopus’s welfare, supporting the researchers in their behavioral studies and avoiding data misinterpretation due to the stress impact of fishing, thus enhancing the quality of physiological and ethological studies.

## Figures and Tables

**Table 1:** Morphometric table used as a measure reference for *O. vulgaris*.

Abbreviation	Measure	Image of the Measure	Definition
dML	Dorsal Mantle Length	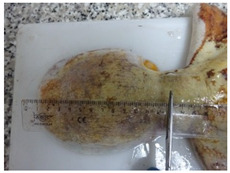	From the midpoint between the eyes to the distal end of the mantle
vML	Ventral Mantle Length	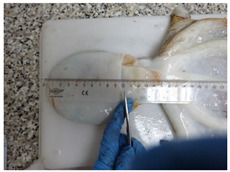	From the midpoint of the mantle edge to its distal end
MW	Mantle Width	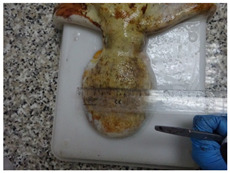	The largest dorsal width of the mantle
HW	Head Width	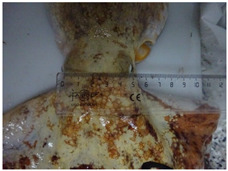	The largest width of head at the eyes level
FuL	Funnel Length	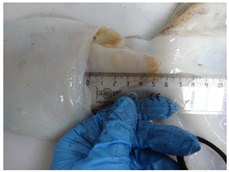	Length of the funnel from the anterior opening to the posterior edge, measured along the ventral midline
TL	Total Length	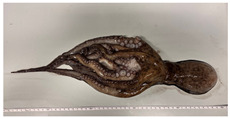	The length from midpoint between eyes to the tip of the arm
TW	Total Weight		Total weight (record made of missing or incomplete arms)
Sex	F/M	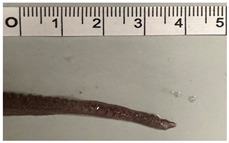	Determined by examining the hectocotylus on the third right arm

**Table 2:** Oligonucleotide primers used in this study.

Primer Name	Primer Sequence (5′–3′)
Ubiquitin—F	TCAAAACCGCCAACTTAACC
Ubiquitin—R	CCTTCATTTGGTCCTTCGTC
Estrogen—F	AACAGCGGCAGATGACATCA
Estrogen—R	GGTGGTGGAGGAGGATGTTG
Catalase—F	CCGTCCCTTTGATAGTTGG
Catalase—R	GGGTCGCCTGTATTCCTAC
Heat Shock Protein 70—F	CCCATAGTTAAGGGGTTGACATC
Heat Shock Protein 70—R	GTGCTTGTTGGTGGCTCTACTAG

**Table 3:** Morphometric data and sex identification of the 29 specimens. The 16 individuals selected for gene expression studies are highlighted (dark gray = fished group; light gray = control group).

ID	Sex	Total Weight (g)	Total Length (cm)	Dorsal Mantle Length (cm)	Ventral Mantle Length (cm)	Mantle Width (cm)	Head Width (cm)	Funnel Length (cm)
P1	F	1010	71.2	16.8	10.5	11.8	3.4	3.5
P2	M	585	48.2	10.2	9.1	9.7	3.4	3.2
P3	M	480	42.7	7.5	7.2	9.3	2.5	2.7
P4	F	1200	62.5	11.6	10.8	11.2	3.2	3.5
P5	M	800	48.6	9,0	8.2	7.8	2.7	3.7
P6	F	1135	61.2	12.7	12.5	13.0	4.2	5.0
P7	F	1210	73.4	13.5	12.9	14.2	3.8	4.5
P8	M	545	48.2	7.4	8.5	9.2	3.1	3.9
P9	M	465	42.8	7.1	7.5	7.8	2.9	3.1
P10	M	940	54.3	11.2	10.5	11.6	3.7	3.9
P11	M	715	49.5	10.9	10.7	11.0	3.3	3.2
P12	F	1075	62.7	12.7	11.5	12.0	3.2	4.5
P13	M	650	38.3	9.9	8.0	10.3	2.2	2.8
P14	M	555	35.5	9.7	7.9	9.2	2.3	2.5
P15	M	1660	69.2	14.3	13.4	13.6	3.6	4.2
P16	F	1335	68.5	12.5	11.7	15.0	4.7	5.5
P17	M	1290	61.1	13.0	12.0	11.5	4.0	5.1
P18	F	1125	77.0	14.0	15.0	12.0	6.0	4.5
P19	F	398	47.0	6.0	7.5	7.0	4.0	2.0
P20	M	149	26.0	5.0	6.5	6.0	3.5	1.5
P21	F	1000	57.0	14.0	10.0	7.0	5.0	4.5
P22	M	500	51.0	8.0	10.0	6.0	3.0	2.0
P23	M	980	67.0	15.0	14.0	10.0	4.0	3.5
P24	M	545	52.0	10.5	10.0	7.5	3.0	2.0
P25	F	985	60.0	12.0	15.0	11.5	4.0	4.0
P26	F	330	51.0	9.0	11.5	7.5	4.0	3.0
P27	F	580	49.0	7.0	7.5	6.5	3.5	2.0
P28	M	950	50.0	12.0	8.5	10.0	5.0	2.5
P29	F	880	64.0	9.0	10.0	9.5	6.0	3.5

## Data Availability

The original contributions presented in this study are included in the article. Further inquiries can be directed to the corresponding author.
